# Development and Validation of Multi-Residue Method for Drugs Analysis in Human Feces by Liquid Chromatography–Tandem Mass Spectrometry

**DOI:** 10.3390/molecules27051474

**Published:** 2022-02-22

**Authors:** Gabriel Míguez-Suárez, Alejandra Cardelle-Cobas, Laura Sinisterra-Loaiza, Beatriz Vázquez, Alberto Cepeda, Carolina Nebot

**Affiliations:** Laboratory of Hygiene, Department of Analytical Chemistry, Nutrition and Bromatology, Faculty of Veterinary Medicine, Universidade de Santiago de Compostela, 27002 Lugo, Spain; gabriel.miguez@rai.usc.es (G.M.-S.); alejandra.cardelle@usc.es (A.C.-C.); laura.sinisterra@usc.es (L.S.-L.); beatriz.vazquez@usc.es (B.V.); alberto.cepeda@usc.es (A.C.)

**Keywords:** antibiotic, food of animal origin, water, feces, food, HPLC–MS/MS

## Abstract

The use of veterinary drugs in animal production is a common practice to secure animal and human health. However, residues of administrated drugs could be present in animal food products. Levels of drugs in food of animal origin are regulated within the European Union. In recent years, residues have been detected not only in food, but also in the environmental elements such as water or soil, meaning that humans are involuntarily exposed to these substances. This article presents a multiclass method for the analysis of various therapeutic groups of pharmaceuticals in human feces. Pharmaceuticals are extracted from feces with an acid extraction solvent, and after filtration the extract was analyzed by HPLC–MS/MS. A limit of detection of 10 ng/g was achieved for 9 pharmaceuticals, with linearity over 0.99 and repeatability and reproducibility lower than 20%. The method was satisfactorily applied in 25 feces samples of individuals that had declared not to be under medical treatment for the last two months. Results indicate the presence of six different compounds at concentration between 10 and 456 ng/g. This preliminary study showed the involuntary exposure of human gut microbiota to active substances such as pharmaceuticals

## 1. Introduction

Direct consumption of pharmaceuticals for the treatment of diseases is inevitable. However, humans are exposed through diet to the consumption of low concentrations of these active compounds in an unintentional way. One route of involuntary intake of pharmaceuticals is the consumption of food of animal origin. Pharmaceuticals can be administrated to animals to treat and prevent infection; according to Pavlov (2008), approximately 80% of animals are treated with pharmaceuticals throughout their lives [1]. Depending on the animal species and the pharmacokinetics of the pharmaceutical, residues of these active compounds in the final food will be different. Maximum residue limits (MRL) of pharmacologically active substances in foodstuffs of animal origin within the European Union are established in the Regulations 37/2010 [2] and 124/2009 [3]. MRL is the maximum concentration of a specific veterinary medicine that can be in food (milk, honey, meat, egg, fish, meat, etc.) expressed in mg/kg or µg/kg (European commission, 2001) [4]. MRLs are calculated on the basis of an estimate amount of the substance present in food, considering a standard shopping basket, the acceptable daily intake (ADI), and which can be consumed daily over a lifetime without appreciable health risk. Therefore, according to the different international organisms, including the European Food Safety Agency, these residues in food must not endanger the health of the consumer.

Vegetables are another route of involuntary consumption of pharmaceuticals. The plants could be treated with drugs to control bacterial infestation. For example, in the United States of America (USA), 0.5% of total antibiotics use were applied to vegetables and fruit, being oxytetracycline and streptomycin the most used [5]. Additionally, if the vegetables are cultivated on soils contaminated with pharmaceuticals, soils fertilized with contaminated manure, or soils irrigated with contaminated water, the plant will adsorb the active compounds. Examples are tetracyclines, sulfonamides and macrolides detected in cabbage, lettuce, carrots, wheat, soybeans, tomatoes, green onions, corn [6,7]. Greenhouse studies with the coccidiostats monensin and lasalocid have also demonstrated that the plant absorbs and accumulates the compounds [8].

Last but not least, water is an involuntary source of pharmaceuticals. The high frequency of their use in human and veterinary medicine, their excretion rate, incomplete removal from wastewater and discharge of contaminant effluent, application of manure with residue of veterinary drugs in agricultural soils as fertilizers, leakage from sewer and septic systems, seepage from rivers and industrial spills all increase the presence of these active compounds into the environment. Once they enter the environment their transport and destiny will depend on factors such as physicochemical properties, environmental conditions (temperature and rainfall) and soil characteristics.

Twenty-one different drugs were detected in water for human consumption, with the presence of anti-inflammatory, antihypertensive and psychiatric [9]. Seventeen pharmaceuticals were also detected in groundwater for human consumption in Galicia, with a concentration ranged between 21 and 14.9 µg/L [10]. Many articles reflect the problem of water contamination with pharmaceuticals, which in many areas are the source of drinking water, and this problem has been described in many countries around the world, including Spain [9,11,12,13,14,15]. The therapeutic groups detected include, among others, antibiotics, antihypertensives, anti-inflammatories, glucocorticoids and antitumoral [11]. In ground water, another source of drinking water, pharmaceuticals have also been detected, and in some countries such as Spain, the frequency of detection reported in groundwater samples is more than 10% [10,16,17]. The frequency of detection changes from one area to another due to different factors such as prescription practices, excretion rate, chemical properties of the compounds, geological characteristics, and weather conditions. It has been demonstrated presence of pharmaceuticals in the environment, and they are entering the food chain through water. A comprehensive research work conducted by Bexfield et al. (2019) monitored 1091 sites of the principal aquifers of the United States, demonstrating the high presence in drinking water of the antibiotic sulfamethoxazole, measured in 23% of the samples [18]. Other studies with fewer sampling points have also demonstrated the presence of antimicrobials in drinking water in Canada [19], Spain [20], Italy [21], Portugal [22], Malaysia [23], China [24]. The antimicrobials most detected in drinking waters are azithromycin, ciprofloxacin, clarithromycin, erythromycin, ofloxacin, sulfadiazine and especially sulfamethoxazole and trimethoprim. Attention is specially paid to the antimicrobial group due to the problems of the development of antimicrobial resistance bacteria which is a world public health issue, as no treatments are available to cure illness caused by these types of bacteria. According to the World Health Organization (WHO), approximately 700,000 people die each year due to drug-resistant diseases such as tuberculosis, sexually transmitted infections, urinary tract infections and respiratory tract infections.

Another problem derived from the consumption of low concentrations of pharmaceuticals is microbiota alteration. It has been seen demonstrated that intake of antibiotics at low doses can alter the gut microbiota [25,26,27,28]. The microbiota could also be altered through proton pomp inhibitors medicines [29].

Many researchers and agencies believe that the concentration of pharmaceuticals involuntarily consumed through the diet is very low and do not treat human health. However, it is important to investigate the level of these active compounds that finally reach the digestive system and not only estimate the concentration through the food basket. Numerous researches on drug toxicity at a high concentration have been published, but the real impact of the continuous exposure over time to low doses of pharmaceuticals and their effects to human health is still unknown. Amoxicillin can be eliminated through feces between 5–10% of the intake doses and it is well known that this drug alters human gut microbiota [25]. Therefore, a first step is to investigate levels of pharmaceuticals that reach the large intestine as an indicator of involuntary exposure to pharmaceuticals. Long-term exposition of human gut microbiota to low doses of pharmaceuticals should be based on data obtained from feces analysis. Therefore, the objective of this article is to present a new method based on HPLC and mass spectrometry detection to evaluate the presence of 24 pharmaceuticals in human feces (Table 1). Up to date and based on the authors knowledge, only one method has been reported to investigate these compounds in the selected matrix [30].

## 2. Results

### 2.1. Optimization of the LC–MS/MS Method

Electrospray ionization was selected as the best choice for the detection of the different pharmaceuticals as available methods for the analysis of these active substances have satisfactory results employing this type of ionization (GC–MS and HPLC–MS analysis of bioactive pharmaceuticals and personal-care products in environmental matrices). Even though, similar values for precursor ion and product ion in different MS instruments are employed, optimization needs to be conducted in the specific instrument that is going to be employed as the mass unit could change from one instrument to another. Standard solutions of individual compounds at 1 μg/L were infused into the MS. Once the precursor ion was selected, collision energy was varied to fragment the analyte and to select product ions with high signal MS response. Selection of precursor and product ions for each of the pharmaceuticals was based on methods previously developed by our research group and those reported by other researchers [31,32,33,34]. To identify each pharmaceutical two product ions and one precursor ion were selected (Table 2), two multiple reaction monitoring (MRM) were employed, the two MRM for identification and one for quantification.

Once MS detection of the pharmaceuticals was optimized, the next step was to obtain a satisfactory chromatographic method where peak shape and resolution should be as high as possible. Even though peak resolution is not the most relevant aspect when triple quadrupole analysis is conducted, it could improve the specificity of the method for compounds with similar chemical properties. On the other hand, Gaussian peak shapes are always recommended for chromatography methods. Pharmaceuticals analysis by HPLC is normally carried out with C18 columns. A wide range of C18 column could be found on the market and the selection would depend on previous experience. A Zorbax eclipse plus from Agilent was employed by Wang et al. (2019) [35] and a Luna C18 from Phenomenex was used for the analysis of 15 pharmaceuticals from wastewater. A complex study was conducted by Cizmic et al. (2017) who compared resolution and peak shapes of 22 pharmaceuticals, among other, azithromycin, sulfamethoxazole, tetracycline, oxtetracycline, ulfadiazine and trimethoprim, achieved with three C18 columns (Synergy fusion, Synergy Hydro and Synergy Polar). The best separation was obtained with the Synergy Polar column [33]. For the analysis of pharmaceuticals present in feces extract three HPLC columns available in the laboratory were tested; an Intensity Solo 2 C18 (100 × 2.1 mm) from Brucker (Bremen, Germany), an Acquity UPLC BEH C18 1,7 µm from Waters (Milford, MI, USA) and a Synergy Polar 5 um (50 × 2.1 mm) from Phenomenex (Torrance, CA, USA). A mixture of various components was tested as mobile phase with each column. The aim was to obtain the highest MS signal response of each of the analytes, analytes separation, peak shapes, and reproducibility. The components tested were water, acetonitrile, methanol with or without formic acid (0.1%), ammonium formate (0.1 mM), oxalic acid (0.1 mM), and ammonium acetate (0.1 M). As similar results were obtained with and without the use of a buffer, it was avoided to reduce salt precipitation in the MS probe. Water and acetonitrile acidified with 0.1% of formic acid were selected as mobile phase components. This mixture was previously reported for the analysis of some of the selected pharmaceuticals [31,32,33,36]. It was very difficult to select between the three tested columns, as very similar results were achieved; however, the Bruker column was selected because the back pressure at the beginning of the analysis with 100% of water phase was lower than with Waters’ column. Additionally, the length of Phenomenex’s column is shorter than the Bruker’s column with the same particle size, and therefore, resolution achieved with the Bruker column was higher.

The method described by Wang et al. (2020) for the analysis of 19 antibiotics in feces samples is laborious as several steps, including solid phase extraction, are required. Therefore, the objective is to present a more simple and cheaper method for the analysis of residues of pharmaceuticals in feces samples [35]. Different solutions and mixtures of solutions were tested for the extraction of the pharmaceuticals from the matrix, among others, methanol, acetonitrile, mixture of acetonitrile and water (MilliQ water), mixture of acetonitrile, water and methanol, 0.1% of formic acid in methanol, 0.1% of formic acid in acetonitrile, 0.1% of formic acid in mixture of acetonitrile and water (MilliQ water), 0.1% of formic acid in a mixture of acetonitrile, water and methanol. QuEChERS extraction with NaCl and MgSO_4_, water and acetonitrile were also investigated. The different extraction protocols were evaluated with four analyte-free replicated samples (0.4 g). One sample was employed as a blank (analyte-free sample) for control, and the other three samples were spiked to 120 ng/g of each pharmaceutical selected for the study. The protocol was tested by adding between 2 and 5 mL of extraction solvent, depending on the method. After shaking and centrifuging the sample, the supernatant was filtered and analyzed by the HPLC–MS/MS with the method developed. The response of the instrument to each pharmaceutical was assessed and compared.

**Figure 1:**
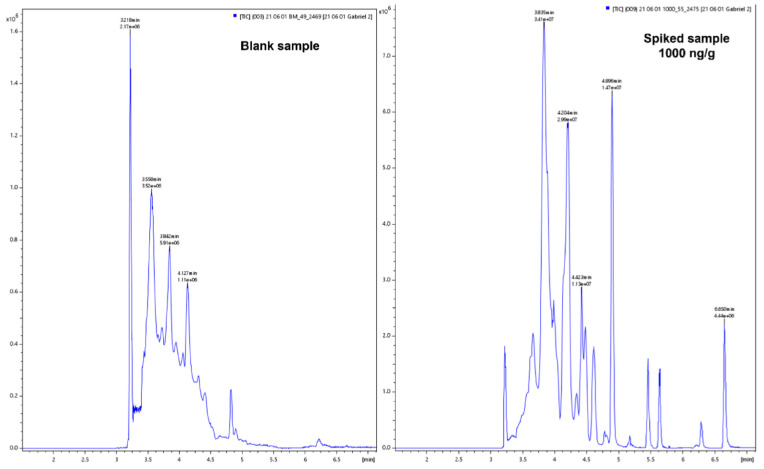
Total ion chromatograms of a blank sample and of a sample spiked with selected pharmaceuticals at 1000 ng/g.

**Figure 2:**
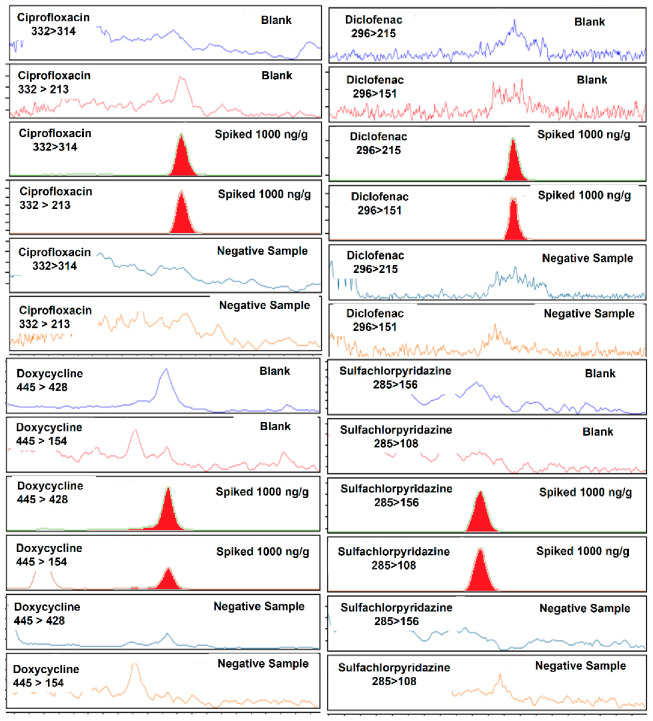
MRM chromatograms of ciprofloxacin, diclofenac, doxycycline and sulfachloropiridazine in a blank sample, sample spiked at 1000 ng/g and in a negative sample.

The use of acetonitrile, methanol or mixed with water (50:50) did not permit the detection of analytes such as danofloxacin, enrofloxacin, some sulfonamides and tetracyclines. Therefore, the use of QuEChERS (NaCl, MgSO_4_, acetonitrile and water) was considered. MgSO_4_ permits higher partition of the pharmaceuticals in the organic phase and the NaCl increases polarity and helps in phase separation. This type of extraction is frequently employed for the extraction of pharmaceuticals, including antibiotics, from food [37] due to the complexity of the matrix, as it reduces considerably the number of steps required during the extraction, as well as the cost of the extraction. For the case of feces samples, more pharmaceuticals were extracted with NaCl and MgSO_4_, acetonitrile and water than with acetonitrile and water without salts. However, the MRM signal for danofloxacin, oxytetracycline and florfenicol were low and the two MRM transitions could not be monitored. The use of formic acid was assessed as follows: formic acid in acetonitrile, in methanol, in a mixture of acetonitrile and water and in a mixture of methanol and water, always at 0.1% of formic acid. Formic acid was selected as it is the most suitable acid for MS analysis. It was observed that its use in a mixture of water: acetonitrile (66:34) improved the recovery of the pharmaceuticals and the number of pharmaceuticals that could be detected. The recoveries and limit of detection achieved with water: acetonitrile was better than those obtained with acidified acetonitrile followed by evaporation as more interference form the matrix was observed. The linearity of method was assessed over a wide range of concentrations from 10 to 2000 ng/g with satisfactory results for most compounds (R^2^ > 0.95). The quantification of some analytes such as azithromycin, dexamethasone and dinofloxacin was discarded during the validation of the method as the results achieved were not acceptable according to the European guidelines (Regulation 2021/808) [38]. Figure 1 shows total ion chromatograms of a blank sample and of an analyte-free sample spiked at 1000 ng/g of each pharmaceutical. Figure 2 shows the two MRM chromatograms employed for ciprofloxacin, diclofenac, doxycycline and sulfachlorpyridazine in a blank sample, the sample spiked at 1000 ng/g of each pharmaceutical and a negative sample was collected from a volunteer.

### 2.2. Method Validation

The limit of quantification (LOQ) is defined as the lowest concentration of pharmaceuticals in a matrix-matched sample spiked with pharmaceuticals which give a signal response higher than 10 in the secondary MRM transition. The LOQ was not the same for all the selected analytes as the instrument response was different depending on the chemical properties of the compounds. For nine analytes the LOQ was 10 ng/g (mefenamic acid, diclofenac, oxytetracycline, sulfachloropyridazine, sulfadimethoxine, sulfamethazine, sulfamethizole, sulfamethoxazole, sulfamethoxypyridazine and trimethoprim) being the maximum LOQ of 250 ng/g for sulfadiazine. According to Wang et al.’s (2020) publication, the LOQ of their method ranged from 0.7 to 4.0 ng/g, while with the method presented the LOQ achieved range between 10 and 250 ng/g. It is important to stand out that while the method presented quantify 24 pharmaceuticals and the one reported by Wang et al., (2020) detects 19 pharmaceuticals, only 9 compounds are included in both methods (Table 3) [35]. It is understandable that the use of a higher number of steps and the use of SPE permitted to achieve lower LOQ; however, it is important to compromise between the cost and time of analysis.

The linearity of the method was also assessed with matrix-matched calibration curves with feces samples spiked with pharmaceuticals from the LOQ to 2000 ng/g. A correlation coefficient above 0.95 is an acceptable value for linearity; the closer the value to one, the better the linearity of the method for the analyte is. For most compounds correlation coefficient was above 0.98, except for the case of clarithromycin, sulfamethazine and sulfamethoxazole as these compounds had correlation coefficient higher than 0.95. 

According to the European Regulation 2021/808, the accuracy of a method, i.e., the closeness of agreement between and the acceptable true reference value, is determined with the trueness and precision [38]. When certified reference materials are not available, the trueness and precision of measurements shall be determined by experiments using fortified blank matrix. Trueness by recovery, repeatability, and within-laboratory reproducibility achieved are summarized in Table 3. Precision under repeatability (RSD_r_) and reproducibility (RSD_R_) conditions results are summarized in Table 3. In general, it could be stated that the presented method shows good precision for antibiotics of the three main groups sulfonamides, tetracyclines and quinolones, which is very important for further evaluation of theirs effects to gut microbiota. The highest RSD values were obtained for reproducibility conditions, as expected, as the experiment is conducted over three different days, clarithromycin, chlortetracycline, danofloxacin, norfloxacin, sulfadiazine and tetracycline showed the higher RSD_R_, above than 10 but below than 20; therefore, values are within an acceptable range (Regulation 2021/808). The RSD reported by Wang et al. (2020) are lower than those achieved with the presented method, and the difference could be due to the number of samples; Wang et al. (2020) only employed three samples to calculate RSD and, in the presented method, a total of six samples were employed. The validation of Wang and collaborators’ method did not follow any specific guideline, while the presented method was validated according to European Regulation applicable for the analysis of pharmaceuticals in food samples. The results achieved with the validation could only be compared with the method reported by Wang et al. (2020) as no other methods for the analysis of the same or similar compounds were found. The results could be compared with methods published for pharmaceuticals in food matrices; however, the compounds and the matrix are different and thus the results are not comparable.

The matrix effect is the effect that the matrix could have on the drug concentration calculation. It is evaluated by comparing the response of the instrument to the compounds dissolved in a solvent to a matrix-matched sample. In this case, feces could affect pharmaceutical concentration by interfering in the extraction and reducing the efficiency. Feces matrix could also interfere the signal response by amplifying or lowering it and consequently increasing or reducing calculated concentration. The matrix factor (MF) for each drug was calculated as the peak area of a matrix-matched standard against the peak area of a solution standard. The results are summarized in Table 3. In general, MF were around one except for the case of mefenamic acid, diclofenac and lincomycin where values were 1.6, 1.7 and 1.4, respectively. The RSD of the MF calculated as the mean of the MF obtained for the concentration range from LOD to 2000 ng/g were in all cases below 20%, which is a satisfactory value according to the European recommendation (Regulation 2021/808).

### 2.3. Application in Feces Samples

The developed method was applied to 25 feces samples obtained from voluntary adults who were not exposed to pharmaceuticals in the two months prior to sample collection, neither by medical prescription nor by voluntary intake. Samples from voluntaries were analyzed following the protocol described previously, and with each batch of samples, six control samples were extracted and analyzed simultaneously for control and quantification.

**Figure 3:**
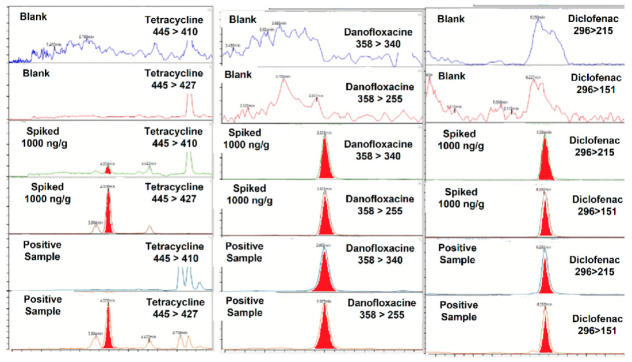
MRM chromatograms of ciprofloxacin, diclofenac, doxycycline and sulfachloropiridazine in a blank, spiked with pharmaceuticals at 1000 ng/g and in positive samples.

Out of 25 feces samples analyzed, six resulted to be positive in the presence of pharmaceuticals. Chlortetracycline, danofloxacin, diclofenac, dinofloxacin, mefenamic acid, sulphaquinoxaline and tetracycline were detected and their concentrations ranged between 10 ng/g of mefenamic acid and 456 ng/g of chlortetracycline. None of the samples contained more than one compound. Figure 3 shows two MRM chromatograms for three positive samples: one to tetracycline (100 ng/g), another to danofloxacin (301 ng/g) and one to diclofenac (86 ng/g).

According to the information facilitated by the volunteers, none of them consumed pharmaceuticals in the two months before feces sample collection. Therefore, pharmaceuticals detected should come from an involuntary intake, certainly from food of animal origin, water or vegetables. None of the positive samples belong to individuals with vegetarian or vegan diets. Voluntaries of the positive samples indicated that the food was bought in large supermarkets, it could be assumed that the food complies the European legislation and the possible presence of residues of pharmaceutical would have been below the MRL (Regulation 37/2010) [2]. In the positive samples, four antibiotics were detected, those substances have MRL in food of animal origin established in the European Regulation 37/2010. The impact of the detected substances to the volunteers’ health is not known. It is important to highlight that alteration of gut microbiota was observed in mice exposed to low levels of antibiotics (ampicillin, sulfadiazine and tetracycline), with an increase of proteobacteria and decreases of *Bifidobacterium* and *Lactobacillus* when low doses of ampicillin and sulphadiazine were suministrated to the animals [39]. Therefore, even if those studies have still not been investigated in humans, an alteration of gut microbiota is expected after long exposure to low doses of antimicrobials. Additionally, microbiota alteration has been related to human health problems and diseases [40]. More research should be conducted to evaluate and determine the effect of the consumption of low doses of antibiotics.

## 3. Materials and Methods

### 3.1. Chemicals, Reagents, and Stock Solutions

Amoxicillin, azithromycin, cefuroxime, chloramphenicol, ciprofloxacin, clarithromycin, colistin, danafloxacin, decoquinate, dexamethasone, diclofenac, difloxacin, doxycycline, enrofloxacin, erythromycin, florfenicol, flumethasone, griseofulvin, ibuprofen, levofloxacin, lincomycin, maduramicin, mefenamic acid, monesin, narasin, nicarbazin, norfloxacin, oxytetracycline, paracetamol, propranolol, robenidine, sarafloxacin, salinomycin, spectinomycin, sulfachloropyridine, sulfadiazine, sulfadimethoxine, sulfamerazine, sulfamethasone, sulfamethoxazole, sulfamethoxypyridazine, sulfapyridine, sulfaquinoxaline, sulfathiazole, tamoxifen, tetracycline, trimethoprim and tylosin with purity between 98 and 101% were bought from Sigma-Aldrich (St. Louis, MO, USA). Acetonitrile (ACN) and methanol (MeOH) (HPLC grade ≥ 99%) were obtained from Acros Organics (Geel, Bergium) and purified water was prepared in the laboratory with a Milli-Q system from Millipore (Burlington, MA, USA).

### 3.2. Preparation of Standard Solutions

To prepare the standard solutions, 10 or 20 mg of each drug was accurately weighed (±0.1 mg) with an analytic balance Ohaus GA200 (Näkiton, Greifensee, Switzerland) and transferred to a 25 mL volumetric flask which contained water, ACN, or MeOH, depending on the substance and its solubility. The individual stock solutions were mixed to prepare a final working standard solution at 5 µg/mL in each pharmaceutical. All solutions were stored at −20 °C for a minimum period of one month.

### 3.3. Equipment

Drugs were extracted and analyzed from the feces samples using the following equipment: MS2 Minishaker vortex mixer (IKA, Staufen, Germany), rotatory shaker RSLAB-9 digital rotisserie (Rogo Sampaic, Wissous, France), laboratory centrifuge Eppendorf 5910 R (Eppendorf, Hamburg, Germany), column HPLC Intensity Solo 2 C18 (100 × 2.1 mm) (Brucker, Bremen, Germany), Acquity UPLC BEH C18 1.7 µm (Waters, Milford, MA, USA), Synergi Polar 5 um (2.1 × 50 mm) (Phenomenex, Torrance, CA, USA). The analysis of the samples extracts was conducted with an Elute UHPLC and mass spectrometer with triple quadrupole EVOQ LC-TQ both from Brucker (Bremen, Germany).

### 3.4. Feces Samples

Twenty-five feces samples were obtained from volunteers participating in the international research project IBEROBDIA. The project was funded by CyTED (918PTE0540) and by the State Research Agency of Spain (PCI2018-093245), it has the approval of the Committee of Ethics of the Galician Health System (SERGAS, Xunta de Galicia), code 2018/270. Within the inclusion-exclusion criteria of this project, one of the requirements was no chronic drug use or consumption, especially of antibiotics, in the last two months. The samples of feces were collected by the volunteers at home and taken to the laboratory within two hours and stored frozen until the analysis

The samples were analyzed as follows: 500 mg (±0.1 mg), were weighed in a 15 mL Falcon tubes and 7.5 mL of extraction solvent was added. The extraction solvent was a mixture of ACN:water (66:34) with 0.1% formic acid. After agitation with a vortex mixed for 10 s, the falcon tubes were transferred to a rotary shaker for 20 min and centrifugated at 4500 rpm for 15 min at 4 °C. Approximately 1 mL of supernatant was filtrated through Acrodisc Syringe Filter (Waters, Milford, CT, USA) and transferred to an HPLC amber vial for analysis.

With each batch of feces samples (*n* = 20), matrix-matched control samples spiked with pharmaceuticals at 0, 10, 25, 50, 100, 250, 500, 750, 1000 and 2000 ng/g were prepared and extracted simultaneously with the samples.

### 3.5. LC–MS/MS Conditions

Samples extracts were analyzed by HPLC–MS/MS system consisting of a Bruker Elute UHPLC coupled to a Bruker EVOQ LC-TQ triple quadruple mass spectrometer (Bremen, Germany). After different tests to obtain the best signal response for each compound chromatography separation was achieved by injecting 15 µL of the sample extract into an Intensity solo 2 C18 (100 × 2.1 mm) column (Bruker, Bremen, Germany). The mobile phase was a mixture on a gradient mode of two components: solvent A was 0.1% formic acid in water and solvent B was 0.1% formic acid in ACN. The flow rate was set at 0.300 mL/min with the following gradient program: 0.0–1.0 min 100% solvent A, 1.0–6.0 min 100% solvent A, 6.0–6.5 min 10% solvent A, 6.5–7.5 min 0% solvent A, 7.5–9.0 min 0% solvent A, and 9.0–15.0 min 100%.

Mass spectrometry detection was performed with positive electrospray (ESI+), except for the case of chloramphenicol and florfenicol where negative ESI was used. Drugs were determined by selecting two multiple reaction monitoring (MRM) and their retention times. The following MS parameters were held constant during the analysis: spray voltage 4800 V for positive ionization and 4500 V for negative. The cone temperature was set up at 300 °C and the flow at 20 psi. The temperature of the heated probe was 500 °C, the nebulizer gas flow at 30 psi and the exhaust gas at 50 psi.

### 3.6. Extraction Method Optimization

Different extraction solvents were tested to extract as many pharmaceuticals as possible from the feces samples. Among others, extraction solvent tested included ACN, MeOH, water, mixture of ACN, MeOH and water, mixture of water and ACN or MeOH and Quecher extraction with the use of a mixture of water and an organic solvent (ACN or MeOH) combined with NaCl and MgSO_4_. The different extraction solvents were tested on three replicated samples spiked with pharmaceuticals at 120 ng/g and a blank sample (analyte-free). In all cases the procedure was as follows: (i) weigh the sample, (ii) spike the sample with pharmaceuticals and homogenate the sample, (iii) leave to stand for 1 h, (iv) add the adequate volume of extraction solvent, (v) shake the sample in a vortex shaker, (vi) 20 min in a rotary shaker at room temperature (between 18 and 23 °C), (vii) 15 min of centrifugation at 4500 rpm at 4 °C, and (viii) filtrate the supernatant through an Acrodisc Syringe Filter (Waters, MA, USA) and transfer to an HPLC amber vial.

A calibration curve of the standard solution of a mixture of pharmaceutical at 0, 10, 25, 50, 100 and 250 ng/mL was prepared and injected also with the resultant extract to evaluate extraction efficiency.

### 3.7. Validation

Validation was conducted following two European guidelines, Regulation 2021/808 that aimed Regulation 2002/657. The following aspects of the method were evaluated: signal noise ratio (S/N), RSD of the Rt, linearity, matrix effects, recovery, precision under repeatability and reproducibility conditions. To validate this method analyte-free feces were spiked to obtain the following concentrations: 0, 10, 25, 50, 100, 250, 500, 750, 1000 y 2000 ng/g. For each concentration, six replicates were employed, and the experiment was repeated on three different days.

## 4. Conclusions

The novelty of the manuscript is to report a new HPLC–MS/MS multiclass method for the identification and quantification of 24 different pharmaceuticals in human feces. The method was satisfactorily validated according to the European Regulation 2021/808 allowing its use and application in reference laboratories of the European Union. The article also presents the results obtained from the application of the method in human feces obtained from individuals that declared not to have taken voluntary pharmaceuticals within the two months previously to the sample collection. Six samples resulted to be positive to six antibiotics and, as health effects are unknown, this work opens a new interesting line of research for future works not only to investigate the effect to gut microbiota but also concentrations of pharmaceuticals in the feces and their relationship with the type of diet.

## Figures and Tables

**Table 1:** Names, therapeutic group, CAS Register Number (CAS), molecular weight (MW) and chemical formula of the selected compounds.

Compound	Therapeutic Groups	CAS	MW	Formula
Mefenamic Acid	Anti-inflammatory	61-68-7	241.28	C_15_H_15_NO_2_
Ciprofloxacin	Antibiotic	85721-33-1	331.34	C_17_H_18_FN_3_O_3_
Clarithromycin	Antibiotic	81103-11-9	747.96	C_38_H_69_NO_13_
Chlortetracycline	Antibiotic	57-62-5	478.88	C_22_H_23_ClN_2_O_8_
Danofloxacin	Antibiotic	112398-08-0	357.38	C_19_H_20_FN_3_O_3_
Diclofenac	Anti-inflammatory	15307-86-5	296.15	C_14_H_11_C_l2_NO_2_
Doxycycline	Antibiotic	564-25-0	444.44	C_22_H_24_N_2_O_8_
Levofloxacin	Antibiotic	100986-85-4	361.37	C_18_H_20_FN_3_O_4_
Lincomycin	Antibiotic	154-21-2	406.54	C_18_H_34_N_2_O_6_S
Norfloxacin	Antibiotic	70458-96-7	319.33	C_16_H_18_FN_3_O_3_
Oxytetracycline	Antibiotic	79-57-2	460.44	C_22_H_24_N_2_O_9_
Sarafloxacin	Antibiotic	98105-99-8	385.36	C_20_H_17_F_2_N_3_O_3_
Sulfachloropyridazine	Antibiotic	80-32-0	284.73	C_10_H_9_Cl-NO_2_S
Sulfadiazine	Antibiotic	68-35-9	250.28	C_10_H_10_N_4_O_2_S
Sulfadimethoxine	Antibiotic	122-11-2	310.33	C_12_H_14_N_4_O_4_S
Sulfamerazine	Antibiotic	127-79-7	264.31	C_11_H_12_N_4_O_2_S
Sulfamethazine	Antibiotic	57-68-1	278.33	C_12_H_14_N_4_O_2_S
Sulfamethoxazole	Antibiotic	723-46-6	253.28	C_10_H_11_N_3_O_3_S
Sulfamethoxypyridazine	Antibiotic	80-35-3	280.3	C_11_H_12_N_4_O_3_S
Sulfapyridine	Antibiotic	144.83-2	249.29	C_11_H_11_N_3_O_2_S
Sulfaquinoxaline	Antibiotic	59-40-5	300.34	C_14_H_12_N_4_O_2_S
Sulfathiazole	Antibiotic	72-14-0	255.32	C_9_H_9_N_3_O_2_S_2_
Tetracycline	Antibiotic	60-54-8	444.43	C_22_H_24_N_2_O_8_
Trimethoprim	Antibiotic	738-70-5	290.32	C_14_H_18_N_4_O_3_

**Table 2:** Retention time (t_R_), multiple reaction monitoring (MRM) 1 and 2 employed for identification and quantification of the pharmaceuticals.

Compound	t_R_ (min)	MRM 1	MRM 2
Mefenamic Acid	6.6	242 > 223	242 > 209
Ciprofloxacin	3.8	332 > 314	332 > 231
Clarithromycin	5.5	749 > 116	749 > 158
Chlortetracycline	4.3	479 > 462	479 > 444
Danofloxacin	3.9	358 > 340	358 > 255
Diclofenac	6.2	296 > 215	296 > 151
Doxycycline	4.4	445 > 428	445 > 154
Levofloxacin	3.9	362 > 261	362 > 179
Lincomycin	3.6	407 > 126	407 > 359
Norfloxacin	3.8	320 > 302	320 > 276
Oxytetracycline	3.9	461 > 443	461 > 426
Sarafloxacin	4.2	400 > 299	400 > 382
Sulfachloropyridazine	4.5	285 > 156	185 > 108
Sulfadiazine	3.9	251 > 156	251 > 108
Sulfadimethoxine	4.9	311 > 156	311 > 108
Sulfamerazine	4.1	265 > 172	265 > 156
Sulfamethazine	4.3	279 > 156	279 > 186
Sulfamethoxazole	4.6	254 > 92	254 > 156
Sulfamethoxypyridazine	4.2	281 > 156	281 > 92
Sulfapyridine	4.0	250 > 156	250 > 92
Sulfaquinoxaline	4.9	301 > 92	301 > 156
Sulfathiazole	3.9	256 > 156	256 > 92
Tetracycline	4.0	445 > 427	445 > 410
Trimethoprim	3.9	291 > 230	291 > 123

**Table 3:** Correlation coefficient (R^2^), limit of quantification (LOQ), trueness, precision under repeatability (RSDr) and reproducibility (RSDR) conditions, and matrix factor of pharmaceuticals selected.

Compound	R^2^	LOQ (ng/g)	Trueness (%)	(Conc) * (ng/g)	RSDr (%)	RSDR (%)	Mean Matrix Factor	Matrix Fractor RSD
Mefenamic Acid	0.995	10	109	50	4.17	3.82	1.60	19.58
Ciprofloxacin	0.995	50	89	50	3.11	5.74	0.91	5.74
Clarithromycin	0.959	25	96	50	8.02	14.24	0.87	10.23
Chlortetracycline	0.994	100	113	100	9.6	15.60	0.77	16.90
Danofloxacin	0.999	25	116	50	3.63	10.82	1.24	9.87
Diclofenac	0.995	10	118	50	3.55	4.50	1.76	19.00
Doxycycline	0.997	25	118	100	4.04	3.55	0.87	16.76
Levofloxacin	0.999	25	116	50	4.54	3.03	0.83	20.13
Lincomycin	0.990	25	91	50	1.17	1.36	1.40	17.24
Norfloxacin	0.998	25	104	250	5.80	19.87	0.83	18.00
Oxytetracycline	0.992	10	112	100	7.79	6.36	0.77	19.51
Sarafloxacin	0.999	25	115	25	1.74	3.40	0.89	14.68
Sulfachloropyridazine	0.998	10	117	50	1.58	2.14	1.01	16.52
Sulfadiazine	0.995	250	93	250	10.99	19.20	0.72	96.16
Sulfadimethoxine	0.999	10	82	50	6.49	2.45	0.99	19.54
Sulfamerazine	0.999	25	110	50	0.89	1.89	0.10	7.40
Sulfamethazine	0.999	10	118	50	1.85	2.56	1.00	12.83
Sulfamethoxazole	0.977	10	111	25	5.78	3.50	1.02	13.19
Sulfamethoxypyridazine	0.999	10	108	50	3.28	3.42	1.02	14.47
Sulfapyridine	0.999	25	118	50	1.93	3.06	1.05	10.11
Sulfaquinoxaline	0.999	25	110	25	6.71	6.87	0.95	17.61
Sulfathiazole	0.999	25	110	50	1.23	3.96	1.27	19.40
Tetracycline	0.995	100	100	25	3.44	10.21	0.98	17.09
Trimethoprim	1000	10	118	50	2.11	2.67	1.08	11.11

* Concentration for which RSDr and RSDR were calculated.

## Data Availability

Not applicable.
